# Chromium Propionate and Zilpaterol Hydrochloride Supplementation in Finishing Lambs: Growth Performance, Body Composition, Carcass Characteristics, and Meat Quality

**DOI:** 10.3390/ani16193068

**Published:** 2026-09-29

**Authors:** Anselmo Hernández-Tovar, Esaúl Hernández-García, Rosalba Lazalde-Cruz, Daniel Rodríguez-Cordero, Pedro Hernández-Briano, Oliver Yaotzin Sánchez-Barbosa, Alejandro Rivera-Villegas, Octavio Carrillo-Muro, Alejandro Plascencia

**Affiliations:** 1 Unidad Académica de Medicina Veterinaria y Zootecnia, Universidad Autónoma de Zacatecas, General Enrique Estrada 98500, Mexico; chemo050@hotmail.com (A.H.-T.); pedro.hernandez@uaz.edu.mx (P.H.-B.); oliver.sanchez@uaz.edu.mx (O.Y.S.-B.); alejandro.rivera@uaz.edu.mx (A.R.-V.); 2 Instituto de Investigaciones en Ciencias Veterinarias, Universidad Autónoma de Baja California, Mexicali 21100, Mexico; esaul.hernandez@uabc.edu.mx (E.H.-G.); rosalba.lazalde@uabc.edu.mx (R.L.-C.); daniel.rodriguez64@uabc.edu.mx (D.R.-C.); 3 Facultad de Medicina Veterinaria y Zootecnia, Universidad Autónoma de Sinaloa, Culiacán 80260, Mexico; alejandro.plascencia@uabc.edu.mx

**Keywords:** adipose deposition, β-adrenergic agonist, dressing percentage, internal fat, *longissimus* muscle area, metabolic modifiers, wholesale cuts

## Abstract

The objective of this study was to evaluate the effects of chromium propionate (Cr-Pr) and zilpaterol hydrochloride (ZH) supplementation on growth performance, performance-derived estimates of dietary energy utilization, body composition, carcass traits, wholesale cut distribution, and meat quality of finishing lambs. Forty intact male Dorper × Katahdin lambs were blocked by initial body weight (38.21 ± 1.72 kg) and assigned to a 2 × 2 factorial arrangement (*n* = 10/treatment): Control (0 Cr-Pr + 0 ZH), ZH (0 Cr-Pr + 7.2 mg ZH/lamb/d), Cr-Pr (1.2 mg elemental Cr/lamb/d + 0 ZH), and Cr-Pr + ZH (1.2 mg elemental Cr/lamb/d + 7.2 mg ZH/lamb/d). The experimental period lasted 45 d; Cr-Pr was supplemented throughout d 1–45, whereas ZH was administered during d 15–42, followed by a 3 d withdrawal period (d 43–45). Under the conditions evaluated, Cr-Pr, ZH, and their interaction did not affect growth performance or performance-derived estimates of dietary energy utilization (*p* > 0.05); however, both compounds produced specific changes in body composition and carcass traits. Supplementation with Cr-Pr reduced omental, visceral, and perirenal fat by 21.8, 18.6, and 18.7%, respectively (*p* ≤ 0.044), and increased *longissimus* muscle area (LMA) by 5.3% (*p* = 0.013). An interaction between Cr-Pr and ZH was detected for rib fat thickness (FAT) (*p* = 0.050), with the ZH and Cr-Pr individual treatments showing lower FAT than Control and Cr-Pr + ZH. Supplementation with ZH increased dressing percentage by 7.2% (*p* = 0.048). Regarding wholesale cuts, classified according to Institutional Meat Purchase Specifications (IMPS), Cr-Pr increased shoulder (IMPS 206) and loin (IMPS 231) proportions by 10.3 and 10.6%, respectively, and reduced shoulder (IMPS 207) by 3.3% (*p* = 0.043), whereas ZH increased breast (IMPS 209) weight by 15.0% and its proportion of cold carcass weight (CCW) by 9.7% (*p* ≤ 0.042). Interactions between Cr-Pr and ZH were also detected for ribs (IMPS 209A) and flank (IMPS 232) weights (*p* ≤ 0.047). Supplementation did not affect meat quality traits (*p* ≥ 0.217). Overall, Cr-Pr was associated with reduced internal fat deposition and increased LMA and loin (IMPS 231) proportion, whereas ZH modified dressing percentage and breast (IMPS 209) weight and proportion of CCW. These responses occurred without changes in growth performance or performance-derived estimates of dietary energy utilization and without detectable changes in the evaluated meat quality traits. The interactions observed for FAT, ribs (IMPS 209A), and flank (IMPS 232) were trait-specific and did not indicate a consistent benefit of the combination.

## Simple Summary

During the final finishing phase, lambs may allocate a greater proportion of nutrients to fat deposition; therefore, strategies that promote body and carcass characteristics of greater productive value are of interest. Chromium propionate (Cr-Pr) and zilpaterol hydrochloride (ZH) act through different metabolic mechanisms and may modify body and carcass composition. Therefore, their individual and combined supplementation was evaluated in finishing lambs. Although Cr-Pr and ZH did not affect growth, feed intake, or performance-derived estimates of dietary energy utilization, both produced specific changes in body composition and carcass traits. Supplementation with Cr-Pr reduced omental, visceral, and perirenal fat by 21.8, 18.6, and 18.7%, respectively, increased *longissimus* muscle area by 5.3%, and increased loin proportion by 10.6%. Supplementation with ZH increased dressing percentage by 7.2% and increased breast weight and proportion by 15.0 and 9.7%, respectively. The combined treatment produced variable responses in subcutaneous fat and some carcass cuts, without a consistent benefit compared with individual supplementation. None of the treatments affected the evaluated meat-quality traits. Overall, Cr-Pr mainly reduced internal fat deposition and increased characteristics of the loin region, whereas ZH mainly modified dressing percentage and the distribution of some carcass cuts.

## 1. Introduction

In intensive lamb finishing systems, improving productive efficiency depends not only on increasing growth rate but also on promoting a body composition with a greater proportion of tissues of productive interest. As animals approach physiological maturity, a greater proportion of nutrients and retained energy is directed toward fat deposition, which has a considerably greater energetic cost than lean tissue accretion and consequently reduces the gain-to-feed ratio (G:F) [1,2]. In this context, metabolic modifiers represent a strategy to alter the composition of gain and tissue distribution, with potential effects on growth performance, body composition, and carcass characteristics [3,4,5,6].

Among metabolic modifiers, β-adrenergic agonists (β-AA) have been widely used during the late finishing phase because of their ability to modify nutrient partitioning and promote lean tissue accretion [7]. In particular, zilpaterol hydrochloride (ZH) has been shown to improve growth performance, dietary energy utilization, and carcass characteristics in finishing lambs [8,9]. Previous studies have reported increases in average daily gain (ADG) ranging from 20.1 to 40.6% and in G:F from 16.5 to 43.3%, although reductions in dry matter intake (DMI) have also been reported [10,11,12]. These effects are associated with activation of β-adrenergic receptors (β-AR) in muscle and adipose tissue, promoting greater protein accretion, increased lipolysis, and reduced lipogenesis, which may favor a greater proportion of muscle tissue and lower fat deposition [7]. However, these responses may also affect some meat quality traits, with changes in cooking loss (CKL) and increases of approximately 10% in Warner–Bratzler shear force (WBSF) being reported [12,13,14]. Thus, the productive and carcass advantages associated with ZH must be considered together with potential effects on meat tenderness and its regulatory status. ZH is authorized for use in finishing cattle in countries including Mexico, the United States, Canada, and South Africa [11]. In the United States, its approved use includes administration during the final 20 to 40 d of finishing followed by a minimum 3-d withdrawal period before slaughter [15], whereas the use of β-AA for growth promotion is prohibited in other markets, including the European Union [16]. From a consumer-safety perspective, compliance with withdrawal periods and residue limits is therefore essential; the Joint FAO/WHO Expert Committee on Food Additives established an acceptable daily intake and recommended maximum residue limits for ZH in edible cattle tissues [17].

Chromium (Cr) also acts as a metabolic modifier, mainly by enhancing insulin signaling and participating in the regulation of carbohydrate, lipid, and protein metabolism [18,19,20]. In cattle, supplementation with Cr propionate (Cr-Pr) has improved insulin sensitivity [21], modified glucose and lipid metabolism [22], and altered the cellular distribution of glucose transporter type 4 (GLUT4) in skeletal muscle, with increased GLUT4 internalization without changes in total GLUT4 content [23]. Taken together, these effects suggest that Cr-Pr may influence nutrient utilization in insulin-sensitive tissues and thereby contribute to changes in body composition. In finishing lambs, Cr-Pr has also shown favorable responses in growth performance, dietary energy utilization, and body composition [24].

Although ZH and Cr-Pr act through different mechanisms, both may modify metabolic processes related to muscle accretion, fat deposition, and body composition; therefore, their combined use could produce independent responses or interactions in these variables. However, information on the combination of both metabolic modifiers in finishing lambs remains limited, particularly regarding their effects on growth performance, performance-derived estimates of dietary energy utilization, body composition, carcass characteristics, wholesale cuts, and meat quality. Therefore, we hypothesized that Cr-Pr and ZH supplementation, individually or in combination, would promote muscle accretion and reduce fat deposition, with potential effects on growth performance, performance-derived estimates of dietary energy utilization, and carcass characteristics. Finishing lambs provide a relevant production model for evaluating these responses because both Cr-Pr and ZH have been individually associated with changes in nutrient utilization and tissue distribution in this species. The objective of this study was to evaluate the effects of Cr-Pr and ZH, as well as their interaction, on growth performance, performance-derived estimates of dietary energy utilization, body composition, carcass characteristics, wholesale cuts, and meat quality of finishing lambs.

## 2. Materials and Methods

The study was conducted at the Experimental Center of Multinutrimentos, Aditivos y Asesoría Zootécnica (MAAZ), located in Calera de Víctor Rosales, Zacatecas, Mexico (22°56′51.23″ N, 102°41′32.33″ W; 2162 m above sea level), a region characterized by a temperate semiarid climate. The 45 d experimental period extended from 27 April to 10 June 2026, and the lambs were slaughtered the following day (11 June 2026) at the slaughter facility of the Unidad Académica de Medicina Veterinaria y Zootecnia, Universidad Autónoma de Zacatecas (UAMVZ-UAZ), located in General Enrique Estrada, Zacatecas, Mexico. At this facility, slaughter and sample collection were performed, whereas carcass and meat quality evaluations were subsequently conducted at the Meat Science and Technology Laboratory of UAMVZ-UAZ. The chemical composition of representative basal-diet samples was determined at the Animal Nutrition Laboratory of the Instituto de Investigaciones en Ciencias Veterinarias, Universidad Autónoma de Baja California.

The experimental protocol was reviewed and approved by the Institutional Committee on Bioethics and Animal Welfare of UAMVZ-UAZ (Protocol #2026/04/18).

### 2.1. Animal Processing, Housing, and Feeding

Forty intact male Dorper × Katahdin lambs, approximately 6 months of age, were purchased specifically for the experiment from the same production unit and selected within the body weight range established for the study. The selected lambs were transported approximately 12 km to the MAAZ Experimental Center following Mexican regulations for animal transportation and humane handling [25,26]. At arrival, each lamb was identified with a numbered ear tag and weighed using an electronic scale (Rhino^®^ BAC-300, 300 kg capacity; Rhino Maquinaria S.A. de C.V., Estado de México, Mexico). As part of the preventive health program, each animal was administered a clostridial bacterin-toxoid at 2.5 mL/lamb subcutaneously (Bovimune^®^ Clostri-10, Lapisa S.A. de C.V., La Piedad, Michoacán, Mexico) and treated against parasites with 1% ivermectin (Ivermectina ATV^®^, ATV Labs, Mexico), according to the dose and route of administration recommended by the manufacturer.

Before the experimental period, lambs were adapted for 21 d to the facilities, management procedures, and finishing diet, including dietary urea. At this age, lambs had a functional rumen capable of utilizing non-protein nitrogen. The dietary transition was achieved by progressively replacing a 50:50 mixture of oat and alfalfa hay with the basal diet until the hay mixture was completely replaced by the end of adaptation. This gradual transition was used to reduce the risk of digestive disorders associated with introducing a high-concentrate diet. At the end of the adaptation period, lambs were individually weighed before the morning feeding to determine initial body weight (IBW; 38.21 ± 1.72 kg, mean ± SD). Based on IBW, animals were ranked and allocated to two blocks of 20 lambs corresponding to the lower and higher IBW ranges. Randomization within each block was performed by drawing labeled slips from a container; five lambs from each block were assigned to each of the four treatments, resulting in 10 lambs per treatment. Pen location was randomly assigned to distribute treatments throughout the experimental area, and animal handling and measurement order were also randomized to minimize potential effects associated with pen location and measurement order. Each lamb was housed in an individual fully covered pen (1.5 × 1.5 m) with an earthen floor and equipped with its own feeder and waterer. Fresh water was available ad libitum throughout the study, and the individually housed lamb was considered the experimental unit. Animal housing, care, and experimental procedures were conducted in accordance with NOM-062-ZOO-1999 [27]. Throughout the 45 d study, animals were monitored twice daily for signs of disease and for digestive or locomotor disorders. A priori humane endpoints for removal from the experiment included marked body weight loss, inability to eat or drink, recumbency, severe disease, severe digestive disorders, or locomotor abnormalities that compromised animal welfare. None of these conditions or other adverse events requiring veterinary intervention or removal from the study occurred. Consequently, all 40 lambs completed the experimental period and were included in the analyses.

### 2.2. Treatments

A 2 × 2 factorial arrangement was used to evaluate the effects of Cr-Pr (0 or 1.2 mg elemental Cr/lamb/d) and ZH (0 or 7.2 mg/lamb/d), with 40 lambs assigned to four treatments (*n* = 10/treatment): Control, ZH, Cr-Pr, and Cr-Pr + ZH. All lambs received the same basal finishing diet. Chromium propionate was supplemented throughout the 45 d experimental period, whereas ZH was administered for 28 d (d 15–42), followed by a 3 d withdrawal period (d 43–45) before slaughter.

The Cr-Pr source was Drescrom P^®^ (Dresen Química, SAPI de C.V., Mexico City, Mexico), containing a minimum of 0.40% Cr (4000 ppm), and was supplied at 0.30 g/lamb/d to provide 1.2 mg elemental Cr/lamb/d. The ZH source was Zilmax^®^ (MSD Salud Animal México, Estado de México, Mexico), supplied at a constant dose of 7.2 mg/lamb/d for 28 d, equivalent to approximately 0.150 mg/kg body weight (BW) based on an expected BW of 48 kg at the midpoint of the supplementation period. The 3 d withdrawal period was consistent with the Mexican product label for finishing cattle [28], whereas the ZH dose and 28 d supplementation period were selected based on previous experimental use of ZH in finishing lambs [6].

Treatment mixtures were prepared in advance using sodium bentonite as a carrier. Each 4 g portion provided half of the daily dose: 0.6 mg elemental Cr, 3.6 mg ZH, or both, according to treatment; the Control received sodium bentonite only. Portions were individually bagged and administered at 08:00 and 16:00 h. Each portion was mixed with approximately 100 g of the basal diet taken from the amount scheduled for the corresponding feeding and therefore did not represent additional feed. This mixture was offered first, and once complete consumption was verified, the remainder of the basal diet scheduled for that feeding was provided. This procedure ensured consumption of the assigned dose and prevented treatment losses in feed refusals. During d 43–45, ZH supplementation was discontinued to comply with the withdrawal period, whereas Cr-Pr supplementation continued through d 45.

The basal finishing diet was formulated to meet the nutrient requirements of finishing lambs according to NRC [1] and was mixed daily in a horizontal paddle mixer. The diet was offered *ad libitum* at 08:00 and 16:00 h, with approximately 40 and 60% of the daily feed allowance provided at each feeding, respectively. Feed refusals were collected and weighed each morning, and feed offered was adjusted to maintain approximately 30 g/kg refusals on an as-fed basis. Individual DMI was calculated daily as the difference between feed offered and refused, expressed on a dry matter (DM) basis. Basal-diet samples were obtained throughout the 45 d period, combined into a composite sample, and refrigerated at 4 °C pending chemical analysis. Ingredients, chemical composition, and calculated net energy (NE) values of the diet are presented in Table 1.

### 2.3. Chemical Analyses

Representative samples of the basal diet were analyzed in triplicate to determine chemical composition. The DM was quantified after drying at 105 °C to constant weight in a forced-air oven (Thermo Scientific, Waltham, MA, USA; AOAC 930.15), whereas ash was quantified by incineration in a muffle furnace (AOAC 942.05). Crude protein was calculated from the nitrogen concentration measured with an FP-528 analyzer (LECO Corp., St. Joseph, MI, USA; AOAC 984.13) [29]. For neutral detergent fiber, samples were analyzed with an Ankom 200 analyzer (Ankom Technology, Macedon, NY, USA) according to Van Soest et al. [31], whereas ether extract was quantified with an Ankom ^XT15^ extractor (Ankom Technology, Macedon, NY, USA) following AOCS method Am 5-04 [30]. Organic matter was calculated as 100 minus ash concentration, whereas starch was estimated from ingredient composition using tabular values from NRC [1].

### 2.4. Growth Performance and Performance-Derived Estimates of Dietary Energy Utilization

Individual BW was recorded before the morning feeding on d 0 and again on d 45 after an 18 h feed withdrawal with free access to water to obtain IBW and FBW, respectively. For performance-derived estimates of dietary energy utilization, ISBW was obtained as IBW × 0.96 to account for gastrointestinal fill [32]. Because the final weighing was performed after the 18 h feed withdrawal, FBW was used directly in the calculations. Average daily gain was calculated as (FBW − IBW)/45 d, DMI as the daily difference between feed offered and refused on a DM basis, and G:F as ADG/DMI. Average SBW was obtained as the mean of ISBW and FBW.

Expected DMI was calculated from observed ADG and average SBW as follows: expected DMI (kg/d) = (EM/NE_m_) + (EG/NE_g_), where EM represents the maintenance energy requirement and EG represents energy gain. The EM term was calculated as 0.056 × SBW^0.75^ [33], whereas EG was estimated as 0.276 × ADG × SBW^0.75^; the coefficient 0.276 was based on a mature BW of 113 kg for intact male Dorper × Katahdin lambs [8]. Dietary NE_m_ and NE_g_ used in these calculations were 1.91 and 1.25 Mcal/kg, respectively, based on NRC [1] ingredient values (Table 1).

Performance-derived NE_m_ was obtained using the quadratic equation described by Zinn et al. [34]: *x* = [−*b* − √(*b*^2^ − 4*ac*)]/(2*c*), where *x* = NE_m_, *a* = −0.41 × EM, *b* = 0.877 × EM + 0.41 × DMI + EG, and *c* = −0.877 × DMI. The corresponding NE_g_ was calculated as 0.877 × NE_m_ − 0.41. For NE_m_ and NE_g_, observed-to-expected ratios were obtained by dividing the performance-derived estimates by their respective tabular dietary values, whereas the DMI ratio was obtained as observed DMI/expected DMI.

### 2.5. Body Fat Reserves and Longissimus Muscle Area

Body fat reserves and muscle development were evaluated by real-time ultrasonography by determining *longissimus* muscle area (LMA, cm^2^), rib fat thickness (FAT, mm), rump fat thickness (RFT, mm), and rump muscle length (RML, cm). The LMA and FAT measurements were obtained between the 12th and 13th ribs, whereas rump measurements were obtained at the P8 site, at the junction of the *biceps femoris* and *gluteus medius* muscles between the ischium and ilium, with the transducer positioned parallel to the vertebral column. Before each evaluation, wool was removed from the measurement sites, the area was cleaned with compressed air, and ultrasound gel was applied as a coupling medium. Measurements were performed at the beginning of the experiment (d 0) and on the day of slaughter, after 18 h of feed withdrawal and before transportation to the slaughter facility, using a real-time ultrasound unit (Aloka Prosound 2, Hitachi Aloka Medical, Tokyo, Japan) equipped with a 3.5 MHz linear transducer. Measurements were performed directly on the real-time ultrasound images using the electronic calipers and measurement functions integrated into the ultrasound unit; therefore, no external image-analysis software was used. The LMA was quantified by tracing the cross-sectional boundary of the *longissimus* muscle, whereas FAT and RFT were measured as the thickness of the subcutaneous fat layer at their respective anatomical sites, and RML was determined as the linear length of the rump muscle visualized at the P8 site. All images were obtained and evaluated by the same trained technician to reduce operator-associated variation.

### 2.6. Empty Body Weight, Non-Carcass Components, and Internal Fat Depots

At the end of the 45 d experimental period, lambs were subjected to 18 h of feed withdrawal with free access to water. After the withdrawal period, preslaughter BW was recorded, and lambs were transported approximately 7.28 km to the UAMVZ-UAZ slaughter facility in accordance with Mexican regulations for animal transportation and humane handling [25,26], where they were slaughtered on the same day in accordance with NOM-033-SAG/ZOO-2014 [35]. During processing, non-carcass components were collected and weighed, including the stomach complex, large intestine, small intestine, skin, limbs, head, heart, lungs, liver, spleen, kidneys, testicles, and internal fat depots. Omental, mesenteric, and perirenal fat were weighed separately, and visceral fat was calculated as the sum of omental and mesenteric fat. The gastrointestinal tract was weighed full and again after manual removal of the digesta to determine gastrointestinal tract contents. Empty body weight (EBW) was calculated as the difference between preslaughter BW and the weight of gastrointestinal tract contents, and non-carcass component and internal fat depot weights were expressed relative to EBW (g/kg).

### 2.7. Carcass Characteristics and Shoulder Tissue Composition

Hot carcass weight (HCW) was recorded immediately after slaughter and before carcass chilling. Carcasses were then maintained at 4 °C for 24 h, after which cold carcass weight (CCW) was determined. Dressing percentage was calculated as (CCW/EBW) × 100, whereas cooling loss was calculated as [(HCW − CCW)/HCW] × 100. At 24 h postmortem, carcass morphometric measurements were obtained using a flexible measuring tape, including carcass length, leg perimeter, and chest depth. In addition, the left shoulder corresponding to Institutional Meat Purchase Specifications (IMPS) 207 [36] was physically dissected to separate and weigh muscle and adipose tissues. Muscle and fat were subsequently expressed as percentages of total shoulder weight, and the muscle:fat ratio was calculated following the procedure described by Luaces et al. [37].

### 2.8. Wholesale Cuts

After chilling, the left carcass side was divided into the forequarter and hindquarter, which were weighed individually before further fabrication. The left carcass side was then fabricated according to the Institutional Meat Purchase Specifications (IMPS) and the guidelines of the North American Meat Processors Association [36]. The forequarter was subdivided into neck, shoulder (IMPS 206), shoulder (IMPS 207), rack (IMPS 204), breast (IMPS 209), and ribs (IMPS 209A), whereas the hindquarter was separated into loin (IMPS 231), flank (IMPS 232), and leg (IMPS 233). The forequarter, hindquarter, and each wholesale cut were weighed individually, and their weights were expressed in kg and as a percentage of one-half of CCW, calculated as cut weight from the left carcass side/(CCW/2) × 100.

### 2.9. Meat Quality Traits

After 24 h of chilling, pH was measured at the level of the second lumbar vertebra using a portable digital pH meter (Hanna Instruments, Woonsocket, RI, USA; Model HI-9025). Thereafter, an approximately 500 g section of the *longissimus* muscle (LM) was collected from the left carcass side and immediately vacuum-packaged and frozen at −20 °C for 14 d, without a refrigerated aging period before freezing. Expressible water (EW) was determined using the Grau and Hamm procedure described by Tsai and Ockerman [38]. For this determination, 300 mg of LM tissue was placed between Whatman No. 1 filter papers positioned between two glass plates (15 × 15 cm) and subjected to a constant load of 10 kg for 20 min. The EW was expressed as the percentage of exudate released relative to the initial sample weight and was calculated as [(final filter-paper weight − initial filter-paper weight)/initial sample weight] × 100.

To determine purge loss (PRL) and cooking loss (CKL), 2 cm thick steaks were obtained from the LM between the 12th rib and the second lumbar vertebra. These steaks were vacuum-packaged and frozen at −20 °C for 14 d without refrigerated aging. Samples were then thawed for 24 h at 4 °C, surface-dried, and weighed. For PRL determination, one steak was cut into 15 × 15 × 30 mm blocks and suspended at 4 °C for 24 h. After 24 h, the bag containing the accumulated exudate was weighed, and PRL was calculated as [(bag + exudate weight − initial bag weight)/initial sample weight] × 100. For CKL determination, a separate steak was vacuum-packaged and heated in a water bath at 80 °C until an internal temperature of 70 °C was reached. After heating, samples were surface-dried while still warm and weighed to determine post-cooking weight. Cooking loss was calculated as [(initial weight − post-cooking weight)/initial weight] × 100.

Warner–Bratzler shear force (WBSF) was determined using 2.54 cm-thick LM steaks thawed for 24 h at 4 °C and cooked on an electric grill (GR2120B, Spectrum Brands, Middleton, WI, USA) until an internal temperature of 70 °C was reached, monitored with a digital thermometer (TP700, Shenzhen TOPRIE Electronics Co., Ltd., Shenzhen, China), according to AMSA [39] recommendations. After cooking, steaks were allowed to cool at room temperature (22 °C) for 4 h. Six cylindrical cores (1.27 cm diameter) were then removed parallel to the muscle fiber direction. The cores were sheared perpendicular to the muscle fibers using a texture analyzer (G-R Manufacturing, New York, NY, USA) equipped with a Warner–Bratzler blade operating at a crosshead speed of 200 mm/min. Peak shear force (kgf) was recorded for each core without normalization by cross-sectional area, and the mean value of the six cores was used as the WBSF value for each sample.

### 2.10. Statistical Analyses

Data were analyzed as a randomized complete block design with a 2 × 2 factorial arrangement, considering Cr-Pr (0 or 1.2 mg Cr/lamb/d) and ZH (0 or 7.2 mg/lamb/d), using the GLM procedure of SAS^®^ OnDemand for Academics: SAS Studio Version 3.82 [40]. Lambs were blocked according to IBW, and each animal constituted the experimental unit. The model included Cr-Pr, ZH, Cr-Pr × ZH, and block. IBW was used as a covariate for FBW, ADG, DMI, G:F, EBW, non-carcass component and internal fat depot weights, HCW, CCW, and wholesale cut weights; for FAT, RFT, RML, and LMA, the corresponding d 0 measurement was used. Proportional variables, percentages, and meat quality traits were analyzed without a covariate. Homogeneity of regression slopes was evaluated by testing covariate × treatment factor interactions, with no evidence of heterogeneous slopes, and residual normality was assessed using the UNIVARIATE procedure. No violations requiring data transformation, exclusion, or alternative statistical procedures were detected. Treatments were coded numerically (1–4). Only the study leader and one student responsible for weighing treatment mixtures knew treatment identity; the latter was not involved in treatment administration. Personnel involved in animal management, treatment administration, and data collection, as well as the person conducting the statistical analyses, were unaware of treatment identity, and analyses were performed using the numerical codes.

Variables including a covariate were analyzed by ANCOVA, whereas the remaining variables were analyzed by ANOVA. Factorial interpretation was based on the main effects of Cr-Pr and ZH and their interaction, and the four treatment combinations were compared using Tukey’s test. Statistical significance was established at *p* ≤ 0.05, and results are presented as least-squares means and SEM. No *a priori* power analysis was performed; 40 lambs (10/treatment combination) were used based on the experimental capacity for individually housed animals and the factorial design. Because no primary outcome was prespecified, responses across the evaluated phenotypic traits were considered exploratory, with particular caution for effects close to the significance threshold.

## 3. Results and Discussion

### 3.1. Growth Performance and Performance-Derived Estimates of Dietary Energy Utilization

Under the conditions evaluated, supplementation with Cr-Pr, ZH, or their interaction did not affect FBW, ADG, DMI, or G:F in finishing lambs (*p* ≥ 0.4669; Table 2). For ZH, this lack of response is generally consistent with studies in which changes in FBW, ADG, or DMI were absent or limited [6,8,41,42], although other studies reported greater ADG and G:F without increases in DMI [43] or dose-dependent responses [12,44]. These differences indicate that the productive response to ZH depends on the magnitude and persistence of β-adrenergic stimulation, which may vary with dose, species, sex, age, genetics, diet, management, and environmental conditions [8,12,43,45]. For example, Aguilera-Soto et al. [41] observed greater ADG during the first weeks, but this response disappeared when the entire experimental period was considered, a pattern compatible with progressive attenuation of β-adrenergic signaling because sustained β-AR stimulation may induce phosphorylation, uncoupling, internalization, and down-regulation [45,46]. Thus, the absence of improvements in ADG and G:F during the 28-d ZH supplementation period used in the present study could be related to an insufficient or transient β-adrenergic response rather than to reduced feed intake, because DMI remained unchanged. However, because β-AR density, signaling, and sensitivity were not evaluated, this mechanism should be considered a biologically plausible explanation rather than a demonstrated cause.

The lack of a productive response to Cr-Pr is also consistent with several previous studies in lambs. Dry matter intake has not been modified by different sources of organic Cr [24,47,48,49], and consistent improvements in growth performance have not been observed with Cr yeast (Cr-Yeast), Cr picolinate (Cr-Pic), Cr methionine (Cr-Met), Cr nicotinate (Cr-Nic), or Cr chloride (CrCl_3_) [47,48,50,51]. In contrast, increases in FBW, ADG, and G:F have been reported with Cr-Pr [24], greater ADG and G:F with Cr-Met [49], and improvements in ADG and G:F with Cr-Yeast under high ambient temperature [52]. This variability indicates that the response to Cr is influenced by source, dose, diet, and physiological status and may become more evident under conditions of greater metabolic demand or stress [47,50,52], although the latter factor cannot be used to explain our results because it was not evaluated. The absence of a productive improvement does not exclude a metabolic response. Kitchalong et al. [47] observed no changes in ADG, DMI, or nitrogen utilization with Cr-Pic, but detected a transiently greater insulin response and lower glucose response, without sustained changes in glucose clearance or insulin kinetics. Therefore, transient changes in glucose–insulin dynamics may occur without resulting in greater growth performance, and because insulin sensitivity and glucose utilization were not evaluated in the present study, the lack of response to Cr-Pr cannot be attributed to a specific metabolic mechanism.

The lack of a productive response also extended to performance-derived estimates of dietary energy utilization. Supplementation with Cr-Pr, ZH, or their interaction did not affect observed NE_m_, observed NE_g_, expected DMI, or the observed-to-expected ratios of NEm, NEg, and DMI (*p* ≥ 0.2667). These estimates allow dietary energy inferred from animal performance to be compared with that expected from diet composition [24,49,53]. When ADG or G:F increases without changes in DMI, greater observed NE_m_ and NE_g_ and observed-to-expected NE ratios may reflect greater apparent energy efficiency [53]. This pattern has been observed with ZH [8,54] and with Cr-Pr or Cr-Met [24,49], although it was not reproduced in the present experiment. Similarly, digestive or metabolic changes induced by Cr do not necessarily result in an overall improvement in energy utilization: Cr-Met increased DM digestibility without affecting ADG or G:F [48], Cr-Pic produced transient glucose and insulin responses without changes in nitrogen balance [47], and Cr-Nic or CrCl_3_ modified specific tissues and wholesale cuts without improving growth performance [50]. Consequently, these results indicate that, under the conditions evaluated, Cr-Pr and ZH did not produce a detectable improvement in performance-derived estimates of dietary energy utilization, even though more specific metabolic or tissue responses may have occurred without being reflected in these overall estimates.

### 3.2. Empty Body Weight, Non-Carcass Component Weights, and Internal Fat Depots

Supplementation with Cr-Pr, ZH, or their interaction did not affect EBW (*p* ≥ 0.0800; Table 3). Complete results for the evaluated non-carcass components and internal fat depots are presented in Table S1. The lack of an effect of ZH agrees with our previous CaPr × ZH study [6], although it differs from Rivera-Villegas et al. [54], who observed greater EBW with ZH. Similarly, Cr-Pr did not affect EBW, in contrast with Rivera-Villegas et al. [24]. Therefore, the changes observed in non-carcass components and adipose depots occurred without detectable changes in EBW and reflect tissue-specific responses rather than a modification of empty body size. Among non-carcass components, ZH reduced large intestine weight by 10.2% (*p* = 0.0413) and increased skin weight by 10.5% (*p* = 0.0259), an effect that remained when expressed relative to EBW, with an increase of 14.5% (*p* = 0.0062). These responses differ from studies in which skin, intestines, and most visceral organs remained unchanged [6,43,54], although changes in specific components have also been reported [12]. In contrast with these specific responses, ZH did not affect internal fat depots, in agreement with studies in which kidney, pelvic, and heart fat remained unchanged [12,43] and, partially, with Guerrero-Bárcena et al. [42], who observed lower greasing without changes in FAT or kidney fat; however, other studies reported reductions in specific adipose depots [8,54]. This variability among depots is biologically plausible because β-adrenergic stimulation can directly activate lipolysis in ovine adipose tissue [55], and other β-AA have reduced indicators of adiposity [56]. However, this response was not expressed in the internal fat depots of lambs in the present study.

In contrast with ZH, supplementation with Cr-Pr consistently reduced internal adiposity by decreasing omental, visceral, and perirenal fat by 21.8, 18.6, and 18.7%, respectively (*p* ≤ 0.0440), and by maintaining lower omental and visceral fat when expressed relative to EBW (*p* ≤ 0.0458). Reductions in different adipose depots have also been observed with other sources of Cr [24,47,48,49,51], although the location and magnitude of the response vary among studies. For example, Mostafa-Tehrani et al. [50] did not detect an overall effect of Cr on internal fat, but within Cr-Nic, 200 µg Cr/kg DM resulted in lower internal fat than higher doses, suggesting that the response may depend on source, dose, and the adipose depot evaluated. From a biological perspective, the lower adiposity observed with Cr-Pr is compatible with changes in lipid metabolism. Lower expression of acetyl-CoA carboxylase 1 in lipogenic tissues has been observed with Cr [57], whereas Cr-Pic reduced non-esterified fatty acids (NEFAs) showed a tendency toward lower FAT, and produced transient changes in glucose–insulin dynamics [47]. However, other Cr sources have not shown consistent responses in glucose, insulin, or NEFA [48], and mechanisms related to insulin sensitivity or metabolic differences among adipose depots remain proposed explanations [50]. Therefore, our results support lower fat accumulation in specific adipose depots with Cr-Pr, but do not allow us to establish whether this response resulted from reduced lipogenesis, changes in lipolysis, insulin sensitivity, or a combination of these pathways. In addition, this reduction occurred without changes in DMI, EBW, ADG, G:F, or performance-derived estimates of dietary energy utilization, a pattern also reported when different Cr sources reduced adiposity without improving growth performance [47,48,50,51]. Consequently, the lower adiposity was not accompanied by reductions in feed intake or growth, although the mechanisms underlying this response cannot be established from the present study. Supplementation with Cr-Pr also reduced skin weight by 8.0% (*p* = 0.0489); because this effect has not been consistent among studies and may vary with Cr source or dose [24,48,50], it should be considered a specific response of the present experiment.

Finally, no interaction between Cr-Pr and ZH was detected for EBW, non-carcass components, or internal fat depots. Therefore, the reduction in internal adiposity was attributable to the main effect of Cr-Pr and was not intensified by ZH. The absence of interaction does not indicate antagonism, but rather that the combination did not produce a detectable complementary response in these variables. Overall, Cr-Pr produced the most consistent tissue response in this section by reducing several internal fat depots, whereas ZH modified only specific non-carcass components. These results support compound-specific responses but not an additive or synergistic effect of Cr-Pr + ZH.

### 3.3. Ultrasound Traits, Carcass Characteristics, and Shoulder Tissue Composition

An interaction between Cr-Pr and ZH was detected for FAT (*p* = 0.0500; Table 4). In the absence of Cr-Pr, ZH reduced FAT from 3.55 to 3.10 mm, whereas Cr-Pr, in the absence of ZH, reduced FAT to 3.14 mm. In contrast, the Cr-Pr + ZH combination resulted in a FAT value of 3.53 mm, similar to Control and greater than those observed with Cr-Pr or ZH administered individually. These results indicate that the reduction in FAT observed with each compound separately was not maintained when both were administered together, reflecting a non-additive response for this trait. The reduction in FAT observed with ZH administered individually agrees with reports in which β-adrenergic agonists reduced subcutaneous fat deposition or adiposity [44,54,56], although other studies have not observed consistent changes in this trait or equivalent measurements [8,12,42,43]. Similarly, the lower FAT observed with Cr-Pr administered individually agrees with studies in which Cr supplementation has been associated with lower subcutaneous fat deposition [24,49,51], although Jin and Zhou [48] did not observe changes in FAT. Overall, these results suggest that Cr-Pr and ZH may reduce FAT when administered individually, whereas this response was not observed when both compounds were combined.

Supplementation with Cr-Pr increased LMA by 5.3% (*p* = 0.0128), independently of ZH. In the treatment-combination comparison, both treatments containing Cr-Pr had greater LMA than Control, whereas ZH was intermediate. This result differs from studies in which different Cr sources did not modify LMA or equivalent measurements [24,47,48,49,51]. Therefore, the increase in LMA represents one of the clearest muscular responses to Cr-Pr in the present study and appears to be specific to the *longissimus* region. In contrast, supplementation with ZH did not modify LMA, in agreement with Estrada-Angulo et al. [8] and Carrillo-Muro et al. [6], although other studies reported increases in LMA [42,43,44,54]. A biological basis exists for a muscular response to ZH because changes in β2-adrenergic receptor messenger RNA abundance and myosin heavy-chain isoforms have been observed [44], as well as greater LMA with other β-AA [56]. However, these responses were not expressed in LMA in the present experiment. Rump muscle length was also unaffected (*p* ≥ 0.5085); therefore, the increase in LMA with Cr-Pr did not extend to all muscular measurements obtained by ultrasound, supporting a regional rather than generalized muscular response.

Among carcass characteristics, supplementation with ZH increased dressing percentage by 7.2%, from 50.79 to 54.46% (*p* = 0.0483), independently of Cr-Pr. This effect agrees with studies reporting greater dressing percentage with ZH [8,42,43,44,54], whereas Cayetano-De-Jesus et al. [12] observed a response in the same direction. However, HCW and CCW were not modified; therefore, the greater dressing percentage did not represent an increase in absolute carcass mass, but rather a greater proportion of EBW represented by CCW. The lack of an effect on HCW and CCW agrees with Estrada-Angulo et al. [8] and Carrillo-Muro et al. [6] for ZH and with studies using different Cr sources [47,48,49,51], although other studies reported greater carcass weights with ZH [43,44,54] or with Cr-Pr [24]. Cooling loss, carcass length, leg perimeter, and chest depth were also unaffected by the treatments (*p* > 0.05). Thus, the effect of ZH on dressing percentage occurred without parallel changes in HCW, CCW, or carcass dimensions and should therefore be interpreted as a change in CCW relative to EBW rather than greater carcass growth.

Shoulder tissue composition also remained unchanged; muscle, fat, and muscle:fat ratio were not affected by Cr-Pr, ZH, or their interaction (*p* > 0.05). This response agrees with Carrillo-Muro et al. [6] for ZH but differs from Rivera-Villegas et al. [54], who observed lower fat and a greater muscle:fat ratio. For Cr, some studies reported greater muscle and lower fat in the shoulder [24,49], whereas Jin and Zhou [48] observed no changes in this region. The absence of changes in shoulder tissue composition, together with the stability of RML, indicates that the increase in LMA with Cr-Pr did not correspond to a uniform muscular response across all evaluated regions.

Overall, Cr-Pr increased LMA, ZH increased dressing percentage, and FAT showed a Cr-Pr × ZH interaction, whereas RFT, RML, HCW, CCW, carcass dimensions, and shoulder tissue composition remained unchanged. These results support region-specific modifications in muscle and carcass traits, but not a generalized increase in carcass mass or a consistent additive or synergistic response to Cr-Pr + ZH.

### 3.4. Wholesale Cut Distribution

Supplementation specifically modified the distribution of some wholesale cuts (Table 5). Complete results for the evaluated wholesale cuts are presented in Table S2. An interaction between Cr-Pr and ZH was detected for ribs (*p* = 0.0466), with ZH or Cr-Pr administered individually resulting in greater weight than Control and the Cr-Pr + ZH combination. An interaction was also observed for flank (*p* = 0.0443), with Cr-Pr + ZH resulting in lower weight than Control and the treatments with ZH or Cr-Pr administered individually. These responses indicate that the effects on both cuts depended on the combination of Cr-Pr and ZH and differ from studies in which ZH did not consistently modify ribs, flank, or most wholesale cuts [12,43,54], as well as from Rivera-Villegas et al. [24], in which Cr-Pr did not modify ribs or flank. Therefore, these interactions should be interpreted as region-specific responses rather than evidence of a complementary advantage of Cr-Pr + ZH.

Independently of Cr-Pr, supplementation with ZH increased breast weight by 15.0% (*p* = 0.0145) and its proportion of CCW by 9.7% (*p* = 0.0421), whereas the remaining wholesale cuts showed no main effect of ZH. This response agrees in direction with Rivera-Villegas et al. [54], who observed greater breast weight, and with other studies in which ZH modified only specific cuts [43], although this effect has not been consistently reproduced [12]. Thus, the response of wholesale cut distribution to ZH was selective and was mainly concentrated in the breast. Differences among muscles in the proportion of type II fibers and β-AR could contribute to this heterogeneity [58,59]; however, these characteristics were not evaluated and therefore do not allow the increase in breast or the interactions observed for ribs and flank to be specifically attributed to a particular β-adrenergic mechanism.

Supplementation with Cr-Pr mainly modified the relative distribution of shoulder and loin (IMPS 231). When expressed as a percentage of CCW, shoulder IMPS 206 and loin increased by 10.3 and 10.6%, respectively (*p* ≤ 0.0416), whereas shoulder IMPS 207 decreased by 3.3% (*p* = 0.0430). The increase in loin is particularly relevant because it agrees with the greater LMA observed previously, providing two independent indicators of greater representation of the *longissimus* region. A regional response has also been reported with other Cr sources: Cr-Met increased rack and loin and reduced leg without modifying shoulder [48], whereas Cr-Nic or CrCl_3_ increased specific limb cuts without uniformly modifying the remainder of the carcass [50]. In contrast, Cr-Pr did not modify loin, shoulder IMPS 206, shoulder IMPS 207, ribs, or flank in Rivera-Villegas et al. [24], in which only rack was reduced. These differences indicate that the response of wholesale cuts to Cr is not uniform and may depend on the source, dose, and anatomical region evaluated. In the present study, the increase in loin occurred without changes in CCW and together with opposite responses in shoulder IMPS 206 and IMPS 207, supporting differences in the relative distribution of specific carcass regions rather than a generalized increase in carcass mass or muscle growth. Although effects of Cr on protein synthesis and insulin sensitivity have been proposed to explain regional changes in the carcass [48,50], these pathways were not directly evaluated. Therefore, the agreement between greater LMA and loin proportion should be considered phenotypic evidence of a regional response of the *longissimus*, without attributing it to a specific mechanism of protein accretion.

Overall, Cr-Pr mainly increased loin and shoulder IMPS 206, ZH increased breast, and the Cr-Pr × ZH interactions for ribs and flank were trait-specific. Because these changes occurred without an increase in CCW and the combination did not show a consistently greater response, the results support compound-specific regional modifications in carcass distribution, but not an additive or synergistic effect of Cr-Pr + ZH.

### 3.5. Meat Quality Traits

Supplementation with Cr-Pr, ZH, or their interaction did not affect pH, PRL, CKL, EW, or WBSF (*p* ≥ 0.2169; Table 6), indicating that the previously observed changes in body composition and carcass characteristics occurred without adversely affecting the evaluated meat quality traits. For Cr-Pr, this lack of response agrees with studies in which different sources of organic Cr did not modify pH, PRL, CKL, or WBSF [24,48,51], although specific responses have been reported, such as greater EW with Cr-Pr [24] or greater pH and CKL with Cr-Met [48]. Likewise, reductions in meat fat or intramuscular fat induced by Cr have not necessarily been accompanied by changes in tenderness or EW [48,51]. Therefore, the response of meat quality traits to Cr appears to depend on the Cr source and the variable evaluated, but our results indicate that Cr-Pr did not compromise EW or tenderness under the conditions of the present study. Although Jin and Zhou [48] proposed that changes in pH could be related to glucose utilization and muscle glycogen, these pathways were not directly evaluated; consequently, the stability of pH observed in the present study does not allow inference of specific changes in postmortem glycolysis.

For ZH, the absence of changes in EW, PRL, and CKL agrees with studies in which water losses remained relatively stable [13,42,60], although Holmer et al. [13] observed greater CKL in the *longissimus lumborum*. The response of tenderness has also been variable: some studies detected no changes in WBSF [42], whereas others reported greater WBSF [13,60], with the magnitude of the response depending on muscle and postmortem aging [13]. Changes in pH and color have also been reported with ZH [12,42], although these responses have not necessarily occurred in parallel with changes in EW or WBSF. In the present study, the simultaneous stability of pH, PRL, CKL, EW, and WBSF indicates that ZH did not produce a detectable deterioration in the evaluated meat quality traits. Although changes in cathepsin activity and lipid metabolism have been reported and participation of the calpain–calpastatin system has been proposed [12,13,42], these mechanisms were not evaluated and are not necessary to explain the absence of effects observed in the present experiment.

Finally, no Cr-Pr × ZH interaction was detected for any meat quality trait. Therefore, the combination did not produce additional adverse effects on pH, PRL, CKL, EW, or WBSF. Overall, no differences in the evaluated meat quality traits were detected with Cr-Pr, ZH, or their combination, indicating that the body and carcass modifications observed in other sections were not accompanied by detectable changes in the evaluated technological meat quality traits. In particular, no treatment-related differences in WBSF were detected under the sample-storage and preparation conditions used in the present study. Because samples were frozen rather than subjected to a defined refrigerated aging period, these WBSF findings should be restricted to the conditions evaluated and should not be extrapolated to general eating quality or commercial tenderness. Nevertheless, other studies have reported specific changes in pH, CKL, or WBSF with Cr or ZH [13,48,60].

Taken together, the results show that Cr-Pr and ZH were associated with specific differences in body and carcass characteristics even though FBW, ADG, DMI, G:F, and performance-derived estimates of dietary energy utilization remained unchanged. In particular, Cr-Pr reduced omental, visceral, and perirenal fat accumulation and increased LMA and loin proportion, whereas ZH increased dressing percentage and breast weight. These treatment-associated differences occurred without detectable changes in pH, PRL, CKL, EW, or WBSF under the conditions evaluated. The Cr-Pr + ZH combination did not show consistent superiority over the individual treatments, and the interactions observed for FAT, ribs, and flank were trait-specific. Overall, the observed effects of Cr-Pr and ZH differed among body composition and carcass traits, whereas their combination did not show a consistent advantage across the evaluated variables. These findings should be interpreted within the doses and supplementation periods evaluated and considering the exploratory nature of the study.

The present study should be interpreted considering several limitations. The relatively small number of experimental units per treatment (*n* = 10) may have limited the ability to detect small treatment responses, particularly for traits with greater biological variability. In addition, multiple phenotypic outcomes were evaluated without a prespecified primary outcome; therefore, isolated effects, particularly those close to the significance threshold, should be interpreted cautiously and considered exploratory. Nutrient partitioning and the metabolic mechanisms potentially underlying the observed changes in internal fat depots and carcass traits were not directly evaluated; consequently, these responses should be considered phenotypic evidence of differences in tissue distribution rather than direct evidence of the underlying mechanisms. Finally, the use of individually housed intact male Dorper × Katahdin lambs, a specific finishing diet, and defined Cr-Pr and ZH doses and supplementation periods may limit extrapolation to other breeds, sexes, feeding systems, or supplementation strategies. Further studies under different production conditions are needed to determine the consistency of these responses.

## 4. Conclusions

Under the conditions of this study, Cr-Pr and ZH selectively modified body and carcass characteristics even though FBW, ADG, DMI, G:F, and performance-derived estimates of dietary energy utilization remained unchanged. In particular, Cr-Pr reduced omental, visceral, and perirenal fat accumulation and increased LMA and loin (IMPS 231) proportion, whereas ZH mainly increased dressing percentage and breast (IMPS 209) weight and proportion. For FAT, Cr-Pr and ZH administered individually resulted in lower values than Control and the Cr-Pr + ZH combination, whereas the interactions observed for ribs (IMPS 209A) and flank (IMPS 232) were trait-specific and did not indicate a consistent advantage of the combination. In addition, these responses occurred without detectable changes in pH, PRL, CKL, EW, or WBSF under the conditions evaluated. Overall, Cr-Pr was associated with lower internal fat accumulation and greater representation of the loin region, whereas ZH modified traits related to carcass dressing; however, neither supplementation nor their combination improved whole-animal performance, and the combination did not show a consistent benefit across the evaluated traits. Given the relatively small sample size and the number of endpoints evaluated, particularly isolated effects close to the significance threshold should be interpreted with caution. Because nutrient partitioning was not directly evaluated, these results should be interpreted as selective phenotypic changes in body and carcass traits rather than direct evidence of nutrient repartitioning.

## Figures and Tables

**Table 1:** Ingredient and nutrient composition and calculated dietary energy values of the basal finishing diet.

Ingredients	% of dietary dry matter
Steam-flaked corn	50.00
Corn stover	22.00
Dried distillers grains with solubles	8.00
Soybean meal, 44% CP	8.00
Cane molasses	7.90
Fertilizer-grade urea (46% N)	0.50
Sodium sesquicarbonate	1.50
Calcium carbonate	1.00
Sodium bentonite	1.00
Premix ^1^	0.10
Chemical composition, % ^2^	
Dry matter	88.10
Organic matter	91.30
Crude protein	14.30
Starch ^3^	36.85
Neutral detergent fiber	19.30
Ether extract	3.49
Ash	8.70
Calculated dietary net energy values ^4^	
Net energy for maintenance, Mcal/kg	1.91
Net energy for gain, Mcal/kg	1.25

^1^ The premix provided per kilogram contained 221.6 mg Co, 125,420 mg Fe, 402.5 mg I, 10,064 mg Mn, 17,078.5 mg Zn, 267 mg Se, and 7813 mg Cu, together with 2,595,000 IU vitamin A, 468,750 IU vitamin D, and 28,000 IU vitamin E. ^2^ Dry matter, crude protein, and ash concentrations were analyzed following AOAC [29] procedures. Ether extract was determined using AOCS Official Method Am 5-04 [30], whereas neutral detergent fiber was analyzed using Ankom methodology (Ankom Technology, Macedon, NY, USA). Dry matter is expressed on an as-fed basis, whereas the remaining chemical components are expressed on a DM basis. Organic matter was calculated as 100 minus ash concentration. ^3^ Starch concentration was estimated from dietary ingredient composition according to NRC [1]. ^4^ Reference net energy values were derived from NRC [1] tabular ingredient data.

**Table 2:** Growth performance and performance-derived estimates of dietary energy utilization of finishing lambs supplemented with chromium propionate (Cr-Pr) and zilpaterol hydrochloride (ZH) (*n* = 40; 10 lambs/treatment).

Item ^1^	Chromium ^2^, mg Cr/Lamb/d				SEM ^3^	Effects (*p*-Value)		
	0.00		1.20			Cr-Pr	ZH	Cr-Pr × ZH
	Zilpaterol Hydrochloride, mg/Lamb/d							
	0.00	7.20	0.00	7.20				
Growth performance								
Body weight, kg								
Initial	38.44	37.80	38.57	38.02	0.493	0.2093	0.7067	0.9310
Final	46.44	46.60	46.37	46.83	0.903	0.6704	0.9152	0.8345
ADG, kg/d	0.182	0.189	0.180	0.193	0.021	0.5205	0.9614	0.8447
DMI, kg/d	1.29	1.28	1.29	1.30	0.044	0.7446	0.5119	0.9869
Gain-to-feed ratio	0.140	0.147	0.140	0.147	0.011	0.5608	0.9024	0.4669
Performance-derived estimates of dietary energy utilization								
Observed NE_m_, Mcal/kg	1.75	1.71	1.76	1.81	0.06	0.3189	0.9347	0.4737
Observed NE_g_, Mcal/kg	1.12	1.09	1.14	1.18	0.05	0.3189	0.9347	0.4737
Expected DMI, kg/d	1.14	1.09	1.11	1.16	0.06	0.7175	0.9900	0.4234
Observed:expected NE_m_	0.92	0.90	0.92	0.95	0.03	0.3189	0.9347	0.4737
Observed:expected NE_g_	0.90	0.87	0.91	0.94	0.04	0.3189	0.9347	0.4737
Observed:expected DMI	1.19	1.20	1.15	1.13	0.05	0.2667	0.9004	0.6800

^1^ ADG = average daily gain; DMI = dry matter intake; NE_m_ = net energy for maintenance; NE_g_ = net energy for gain. ^2^ Cr = elemental chromium supplied as chromium propionate (Drescrom P^®^); 300 mg of commercial product provided 1.2 mg Cr/lamb/d. ^3^ SEM = standard error of the mean.

**Table 3:** Empty body weight and selected non-carcass components and internal fat depots of finishing lambs supplemented with chromium propionate (Cr-Pr) and zilpaterol hydrochloride (ZH) (*n* = 40; 10 lambs/treatment).

Item	Chromium ^1^, mg Cr/Lamb/d				SEM ^2^	Effects (*p*-Value)		
	0.00		1.20			Cr-Pr	ZH	Cr-Pr × ZH
	Zilpaterol Hydrochloride, mg/Lamb/d							
	0.00	7.20	0.00	7.20				
Empty body weight, kg	45.71	44.25	45.53	43.69	0.91	0.6856	0.08	0.8387
Non-carcass component and internal fat depot weights, kg								
Large intestine	0.64	0.59	0.64	0.56	0.04	0.6710	0.0413	0.6695
Skin	5.74 ^ab^	6.45 ^a^	5.38 ^b^	5.84 ^ab^	0.25	0.0489	0.0259	0.6215
Omental fat	0.91 ^a^	0.79 ^a^	0.64 ^b^	0.69 ^b^	0.09	0.0321	0.6656	0.3250
Visceral fat	1.37 ^a^	1.27 ^a^	1.05 ^b^	1.10 ^b^	0.10	0.0167	0.8049	0.4723
Perirenal fat	0.58 ^a^	0.49 ^a^	0.43 ^b^	0.44 ^b^	0.06	0.0440	0.5208	0.4444
Non-carcass components and internal fat depots, g/kg empty body weight								
Skin	128.24 ^ab^	147.30 ^a^	120.94 ^b^	138.00 ^ab^	6.10	0.1846	0.0062	0.8714
Omental fat	20.20 ^a^	17.59 ^a^	14.14 ^b^	16.40 ^b^	2.10	0.0458	0.9344	0.2574
Visceral fat	30.73 ^a^	28.69 ^a^	23.24 ^b^	25.66 ^b^	2.30	0.0300	0.9353	0.3393

^1^ Cr = elemental chromium supplied as chromium propionate (Drescrom P^®^); 300 mg of commercial product provided 1.2 mg Cr/lamb/d. ^2^ SEM = standard error of the mean. ^a,b^ Means within a row without a common superscript differ (*p* ≤ 0.05) according to Tukey’s test. Complete results for all evaluated non-carcass components and internal fat depots are provided in Table S1.

**Table 4:** Ultrasound traits, carcass characteristics, and shoulder tissue composition of finishing lambs supplemented with chromium propionate (Cr-Pr) and zilpaterol hydrochloride (ZH) (*n* = 40; 10 lambs/treatment).

Item ^1^	Chromium ^2^, mg Cr/Lamb/d				SEM ^3^	Effects (*p*-Value)		
	0.00		1.20			Cr-Pr	ZH	Cr-Pr × ZH
	Zilpaterol Hydrochloride, mg/Lamb/d							
	0.00	7.20	0.00	7.20				
Ultrasound traits								
Rib fat thickness, mm	3.55 ^a^	3.10 ^b^	3.14 ^b^	3.53 ^a^	0.21	0.9607	0.8602	0.0500
Rump fat thickness, mm	3.13	3.09	2.99	3.09	0.18	0.7071	0.8560	0.6933
Rump muscle length, cm	6.69	6.92	6.79	6.61	0.30	0.7267	0.9441	0.5085
*Longissimus* muscle area, cm^2^	12.96 ^b^	13.84 ^ab^	14.13 ^a^	14.08 ^a^	0.26	0.0128	0.0898	0.1296
Carcass characteristics								
HCW, kg	24.00	24.43	24.53	24.26	0.55	0.7411	0.8873	0.5345
CCW, kg	23.30	23.53	23.58	23.24	0.58	0.9946	0.9274	0.6300
Dressing, % EBW	50.87 ^b^	54.01 ^a^	50.71 ^b^	54.90 ^a^	1.85	0.8441	0.0483	0.7791
Cooling loss, %	3.51	3.21	3.24	3.45	0.26	0.9591	0.8723	0.3377
Carcass length, cm	81.19	82.29	81.94	80.29	0.92	0.5037	0.7622	0.1444
Leg perimeter, cm	38.25	37.00	39.33	37.14	1.16	0.6020	0.1503	0.6888
Chest depth, cm	19.25	19.64	19.33	19.14	0.56	0.7145	0.8588	0.6089
Shoulder (IMPS 207) tissue composition, %								
Muscle	57.53	61.24	60.60	60.42	1.21	0.3620	0.1561	0.1208
Fat	24.37	21.44	20.95	21.30	1.26	0.1678	0.3125	0.2019
Muscle:fat ratio	2.41	2.86	2.91	2.87	0.22	0.9371	0.2470	0.3845

^1^ FAT = rib fat thickness; RFT = rump fat thickness; RML = rump muscle length; LMA = *longissimus* muscle area; HCW = hot carcass weight; CCW = cold carcass weight; EBW = Empty body weight. ^2^ Cr = elemental chromium supplied as chromium propionate (Drescrom P^®^); 300 mg of commercial product provided 1.2 mg Cr/lamb/d. ^3^ SEM = standard error of the mean. ^a,b^ Means within a row without a common superscript differ (*p* ≤ 0.05) according to Tukey’s test.

**Table 5:** Selected wholesale cut weights and proportions of finishing lambs supplemented with chromium propionate (Cr-Pr) and zilpaterol hydrochloride (ZH) (*n* = 40; 10 lambs/treatment).

Item ^1^	Chromium ^2^, mg Cr/Lamb/d				SEM ^3^	Effects (*p*-Value)		
	0.00		1.20			Cr-Pr	ZH	Cr-Pr × ZH
	Zilpaterol Hydrochloride, mg/Lamb/d							
	0.00	7.20	0.00	7.20				
Breast (IMPS 209), kg	0.78	0.88	0.75	0.88	0.04	0.7234	0.0145	0.7300
Ribs (IMPS 209A), kg	0.78 ^b^	0.87 ^a^	0.84 ^a^	0.72 ^b^	0.05	0.3132	0.8613	0.0466
Flank (IMPS 232), kg	0.95 ^a^	1.00 ^a^	0.94 ^a^	0.76 ^b^	0.06	0.0475	0.2830	0.0443
Shoulder (IMPS 206), % of CCW/2	8.90	8.06	9.12	9.58	0.45	0.0416	0.6713	0.1554
Shoulder (IMPS 207), % of CCW/2	18.46	18.37	17.82	17.80	0.34	0.0430	0.8858	0.9227
Breast (IMPS 209), % of CCW/2	3.41	3.57	3.20	3.68	0.18	0.7926	0.0421	0.3805
Loin (IMPS 231), % of CCW/2	9.84 ^b^	10.18 ^ab^	10.71 ^a^	11.43 ^a^	0.36	0.0068	0.1526	0.5989

^1^ IMPS = Institutional Meat Purchase Specifications; CCW = cold carcass weight. ^2^ Cr = elemental chromium supplied as chromium propionate (Drescrom P^®^); 300 mg of commercial product provided 1.2 mg Cr/lamb/d. ^3^ SEM = standard error of the mean. ^a,b^ Means within a row without a common superscript differ (*p* ≤ 0.05) according to Tukey’s test. Complete results for all evaluated wholesale cuts are provided in Table S2.

**Table 6:** Meat quality traits of finishing lambs supplemented with chromium propionate (Cr-Pr) and zilpaterol hydrochloride (ZH) (*n* = 40; 10 lambs/treatment).

Item	Chromium ^1^, mg Cr/Lamb/d				SEM ^2^	Effects (*p*-Value)		
	0.00		1.20			Cr-Pr	ZH	Cr-Pr × ZH
	Zilpaterol Hydrochloride, mg/Lamb/d							
	0.00	7.20	0.00	7.20				
pH	5.61	5.55	5.58	5.52	0.06	0.6414	0.3181	0.9417
Purge loss, %	4.30	3.44	3.25	3.52	0.44	0.2821	0.5069	0.2169
Cooking loss, %	25.11	25.71	23.00	24.14	3.22	0.5752	0.7902	0.9346
Expressible water, %	33.11	33.75	32.86	33.20	1.44	0.7843	0.7384	0.9203
Warner–Bratzler shear force, kgf	3.40	3.39	3.44	3.75	0.37	0.5937	0.6864	0.6665

^1^ Cr = elemental chromium supplied as chromium propionate (Drescrom P^®^); 300 mg of commercial product provided 1.2 mg Cr/lamb/d. ^2^ SEM = standard error of the mean.

## Data Availability

The data presented in this study are available from the corresponding author upon reasonable request.

## Supplementary Materials

The following supporting information can be downloaded at: https://www.mdpi.com/article/10.3390/ani16193068/s1. Table S1. Empty body weight, non-carcass component weights, and internal fat depots of finishing lambs supplemented with chromium propionate (Cr-Pr) and zilpaterol hydrochloride (ZH); Table S2. Wholesale cut weights and proportions of finishing lambs supplemented with chromium propionate (Cr-Pr) and zilpaterol hydrochloride (ZH).
