# Supplementary material for: Chromium Propionate and Zilpaterol Hydrochloride Supplementation in Finishing Lambs: Growth Performance, Body Composition, Carcass Characteristics, and Meat Quality
Source: Animals (Basel). 2026 Sep 29;16(19):3068. doi: 10.3390/ani16193068 (PMC13663348; doi:10.3390/ani16193068)
Supplement: Supplementary file 1 [file animals-16-03068-s001.zip › animals-4576825-supplementary.pdf]

Supplementary Materials

Chromium Propionate and Zilpaterol Hydrochloride Supplementation in Finishing Lambs: Growth Performance, Body Composition, Carcass Characteristics, and Meat Quality

Table S1. Empty body weight, non-carcass component weights, and internal fat depots of finishing lambs supplemented with chromium propionate (Cr-Pr) and zilpaterol hydrochloride (ZH) (*n* = 40; 10 lambs/treatment).

Item	Chromium[1], mg Cr/lamb/d				SEM [2]	Effects (*p*-value)		
	0.00		1.20			Cr-Pr	ZH	Cr-Pr × ZH
	Zilpaterol hydrochloride, mg/lamb/d							
	0.00	7.20	0.00	7.20				
Empty body weight, kg	45.71	44.25	45.53	43.69	0.91	0.6856	0.0800	0.8387
**Non-carcass component and internal fat depot weights, kg**								
Stomach complex	1.45	1.56	1.48	1.46	0.06	0.5504	0.4567	0.3240
Large intestine	0.64	0.59	0.64	0.56	0.04	0.6710	0.0413	0.6695
Small intestine	0.74	0.74	0.77	0.67	0.04	0.6733	0.2502	0.2545
Skin	5.74[ab]	6.45[a]	5.38[b]	5.84[ab]	0.25	0.0489	0.0259	0.6215
Limbs	0.90	0.99	0.93	0.94	0.05	0.9000	0.2805	0.3578
Head	1.65	1.75	1.73	1.73	0.04	0.3875	0.2205	0.2392
Heart	0.20	0.20	0.22	0.20	0.01	0.3886	0.2710	0.1938
Lungs	0.92	0.95	0.97	1.03	0.05	0.1832	0.2927	0.7109
Liver	0.87	0.87	0.91	0.87	0.03	0.5417	0.4518	0.5452
Spleen	0.09	0.09	0.10	0.08	0.01	0.8552	0.2072	0.2629
Kidney	0.11	0.12	0.11	0.11	0.01	0.3985	0.4408	0.9511
Testicles	0.67	0.68	0.68	0.66	0.04	0.9336	0.8476	0.7521
Omental fat	0.91[a]	0.79[a]	0.64[b]	0.69[b]	0.09	0.0321	0.6656	0.3250
Mesenteric fat	0.47	0.47	0.39	0.41	0.04	0.1343	0.8308	0.8523
Visceral fat	1.37[a]	1.27[a]	1.05[b]	1.10[b]	0.10	0.0167	0.8049	0.4723
Perirenal fat	0.58[a]	0.49[a]	0.43[b]	0.44[b]	0.06	0.0440	0.5208	0.4444
**Non-carcass components and internal fat depots, g/kg empty body weight**								
Stomach complex	32.19	35.12	32.37	34.14	1.67	0.8120	0.1699	0.7333
Large intestine	13.88	12.69	13.95	12.84	0.88	0.9041	0.2054	0.9663
Small intestine	16.22	16.34	17.04	15.66	1.02	0.9440	0.5427	0.4686
Skin	128.24[ab]	147.30[a]	120.94[b]	138.00[ab]	6.10	0.1846	0.0062	0.8714
Limbs	20.04	22.78	20.88	22.16	1.37	0.9353	0.1558	0.5984
Head	36.83	39.74	38.39	39.44	1.39	0.6517	0.1634	0.5071
Heart	4.51	4.60	4.83	4.62	0.25	0.4976	0.8184	0.5484
Lungs	20.28	21.10	20.85	24.11	1.30	0.1797	0.1282	0.3562
Liver	19.60	19.87	19.90	20.26	0.69	0.6210	0.6544	0.9508
Spleen	2.08	2.13	2.21	2.00	0.17	0.9767	0.6350	0.4600
Kidney	2.45	2.64	2.32	2.60	0.17	0.6223	0.1762	0.7997
Testicles	15.15	14.91	14.88	15.52	1.01	0.8688	0.8439	0.6708
Omental fat	20.20[a]	17.59[a]	14.14[b]	16.40[b]	2.10	0.0458	0.9344	0.2574

Mesenteric fat	10.54	11.09	9.10	9.26	1.15	0.1657	0.7540	0.8660
Visceral fat	30.73^{a}	28.69^{a}	23.24^{b}	25.66^{b}	2.30	0.0300	0.9353	0.3393
Perirenal fat	12.73	10.49	9.48	10.25	1.38	0.2173	0.5998	0.2872

[1] Cr = elemental chromium supplied as chromium propionate (Drescrom P®); 300 mg of commercial product provided 1.2 mg Cr/lamb/d. [2] SEM = standard error of the mean. [a,b] Means within a row without a common superscript differ ($p \leq 0.05$) according to Tukey's test.

Table S2. Wholesale cut weights and proportions of finishing lambs supplemented with chromium propionate (Cr-Pr) and zilpaterol hydrochloride (ZH) (n = 40; 10 lambs/treatment).

Item [1]	Chromium[2], mg Cr/lamb/d				SEM [3]	Effects (p-value)		
	0.00		1.20			Cr-Pr	ZH	Cr-Pr × ZH
	Zilpaterol hydrochloride, mg/lamb/d							
	0.00	7.20	0.00	7.20				
**Wholesale cut weights, kg**								
Forequarter	6.35	6.45	6.43	6.37	0.18	0.9882	0.9125	0.6481
Hindquarter	5.41	5.54	5.58	5.29	0.15	0.8025	0.6159	0.1758
Neck	0.88	0.89	0.90	0.90	0.05	0.7747	0.8226	0.9243
Shoulder (IMPS 206)	1.03	0.95	1.08	1.09	0.06	0.1240	0.5754	0.4121
Shoulder (IMPS 207)	2.14	2.12	2.10	2.05	0.06	0.4152	0.5704	0.8064
Rack (IMPS 204)	0.74	0.75	0.78	0.73	0.03	0.8135	0.4862	0.3481
Breast (IMPS 209)	0.78	0.88	0.75	0.88	0.04	0.7234	0.0145	0.7300
Ribs (IMPS 209A)	0.78^{b}	0.87^{a}	0.84^{a}	0.72^{b}	0.05	0.3132	0.8613	0.0466
Loin (IMPS 231)	1.18	1.18	1.27	1.27	0.06	0.1659	0.9593	0.9847
Flank (IMPS 232)	0.95^{a}	1.00^{a}	0.94^{a}	0.76^{b}	0.06	0.0475	0.2830	0.0443
Leg (IMPS 233)	3.28	3.36	3.37	3.26	0.08	0.9767	0.8720	0.2503
**Wholesale cuts, % of cold carcass weight/2**								
Forequarter	54.70	54.49	54.55	55.71	0.83	0.5260	0.5704	0.4142
Hindquarter	46.20	46.88	47.28	46.74	0.53	0.3752	0.8942	0.2548
Neck	3.81	3.73	3.83	4.08	0.23	0.4333	0.7046	0.4669
Shoulder (IMPS 206)	8.90	8.06	9.12	9.58	0.45	0.0416	0.6713	0.1554
Shoulder (IMPS 207)	18.46	18.37	17.82	17.80	0.34	0.0430	0.8858	0.9227
Rack (IMPS 204)	6.29	6.33	6.61	6.34	0.19	0.3996	0.5592	0.4229
Breast (IMPS 209)	3.41	3.57	3.20	3.68	0.18	0.7926	0.0421	0.3805
Ribs (IMPS 209A)	6.61	7.13	6.95	6.46	0.31	0.5970	0.9605	0.1161
Loin (IMPS 231)	9.84^{b}	10.18^{ab}	10.71^{a}	11.43^{a}	0.36	0.0068	0.1526	0.5989
Flank (IMPS 232)	8.00	8.33	7.95	7.32	0.38	0.1690	0.6872	0.2145
Leg (IMPS 233)	28.35	28.37	28.62	27.99	0.38	0.8873	0.4218	0.3964

[1] IMPS = Institutional Meat Purchase Specifications. [2] Cr = elemental chromium supplied as chromium propionate (Drescrom P®); 300 mg of commercial product provided 1.2 mg Cr/lamb/d. [3] SEM = standard error of the mean. [a,b] Means within a row without a common superscript differ ($p \leq 0.05$) according to Tukey's test.
